# Pre-selection of most significant SNPS for the estimation of genomic breeding values

**DOI:** 10.1186/1753-6561-3-s1-s14

**Published:** 2009-02-23

**Authors:** Nicolò PP Macciotta, Giustino Gaspa, Roberto Steri, Camillo Pieramati, Paolo Carnier, Corrado Dimauro

**Affiliations:** 1 Dipartimento di Scienze Zootecniche, Università di Sassari, Italy; 2 Centro di Studio del Cavallo Sportivo, Università di Perugia, Italy; 3 Dipartimento di Scienze Animali, Università di Padova, Italy

## Abstract

The availability of a large amount of SNP markers throughout the genome of different livestock species offers the opportunity to estimate genomic breeding values (GEBVs). However, the estimation of many effects in a data set of limited size represent a severe statistical problem. A pre-selection of SNPS based on single regression may provide a reasonable compromise between accuracy of results, number of independent variables to be considered and computing requirements.

A total of 595 and 618 SNPS were pre-selected using a simple linear regression for each SNP, based on phenotypes or polygenic EBVs, respectively, with an average distance of 9–10 cM between them. Chromosome four had the largest frequency of selected SNPS. Average correlations between GEBVs and TBVs were about 0.82 and 0.73 for the TRAINING generations when phenotypes or polygenic EBVs were considered as dependent variable, whereas they tend to decrease to 0.66 and 0.54 for the PREDICTION generations. The pre-selection of SNPs using the phenotypes as dependent variable together with a BLUP estimation of marker genotype effects using a variance contribution of each marker equal to σ^2^_a_/n_snps _resulted in a remarkable accuracy of GEBV estimation (0.77) in the PREDICTION generations.

## Background

The availability of a large amount of SNP markers throughout the genome for several livestock species allows the prediction of genomic breeding values (GEBVs) as the sum of the effect of the different haplotype intervals that cover the whole genome [1-3]. Although a dense maker map results in a great advantage for identifying genome regions involved in the determinism of a trait, the estimation of the effect of a large number of haplotypes (up to some hundred thousands) based on a limited number of phenotypes (some hundreds) represents a relevant statistical and computational issue. BLUP methodologies are able to predict more haplotype effects than data points by treating them as random and assuming an equal variance for each interval [1]. Furthermore, the reconstruction of parental haplotypes can be avoided by using SNP genotypes directly. Actually, the use of single markers instead of haplotypes resulted in a slight reduction of accuracy in QTL fine mapping [4] and in no sensible differences in MAS accuracy for a low heritability trait [2]. A further issue is whether or not all SNPs should be included in a predictive model [5]. A pre-selection of SNPs based on single regression could represent a reasonable option to speed up calculations. Meuwissen et al. [1] reported an overestimation of haplotype effects and low accuracy of GEBVs when a least-square stepwise regression approach was used to both pre-select SNPs and estimate their effects. On the contrary, in genome wide association analysis, the regression-based pre-selection of SNPs yielded a reasonable statistical power in QTL detection [6]. In any case, considering the size of available SNP platforms (50 k for cattle), a pre-selection of SNPs combined with a BLUP estimation of marker effects could represent an acceptable compromise between accuracy of GEBVs, number of independent variables to be considered and computing requirements. In the present paper, genomic breeding values were estimated on a simulated data set of 5,865 individuals by first selecting the most relevant SNPs and then using a BLUP methodology to estimate marker effects.

## Methods

### Data

A simulated data set of 5,865 individuals generated for the XII QTL-MAS workshop was used. The genome consisted of six chromosomes, with a total of 6,000 SNP marker loci (1,000 per chromosome). Individuals of the first four generations (TRAINING data set) had pedigree, phenotype, and marker information available whereas those of the last three generations (PREDICTION data set) had only pedigree and marker information.

### Polygenic breeding value prediction

Variance components and polygenic breeding values were estimated by analyzing the whole data set with the following single trait BLUP animal model using the MTDFREML package:

y_ijk _= SEX_i _+ GEN_j _+ a_k _+ e_ijk_

where y is the trait value, SEX is the fixed effect of sex (i = 1,2), GEN is the fixed effect of generation (j = 0–6), a_k _is the random genetic additive effect of the k-th animal (**a**~**N**(0, **A**σ^2^_a_)), e_ijk _is the random residual (**e**~N(**0**, **I**σ^2^_e_)). The relationship matrix included 5,939 animals.

### Pre-selection of SNPS

Data of the 4,665 animals of the TRAINING data set were analysed by a simple linear regression for each SNP.

y = μ+ SNP_i _+ e

where y is the phenotype or the polygenic EBV, SNP is the genotype at the i-th SNP (i: 1 to 6,000). An empirical threshold of 1.6E^-6 ^for the *P *values of the F test was fixed to retain markers for further multiple-SNP analysis.

### GEBVs estimation

The effect of marker genotypes was estimated on the TRAINING data set with the following mixed linear model

$$\text{y}=\mu +{\text{SEX}}_{\text{i}}+{\text{GEN}}_{\text{i}}+\sum_{k=1}^{m}H_{k}b_{k}+\text{e}$$

where y is the phenotype, **b **is a vector of three genotype effects for SNP k, and **H **is the corresponding design matrix for the SNP k, and e is the random residual. No interaction between different SNPs loci and a constant variance for each SNP locus were assumed. In studies where no variance components are estimated, the variance ratio λ = σ^2^_e_/σ^2^_a _to be used in the solution of mixed model equations is usually fixed a priori at a value (for example 1) able to remove possible dependencies among intervals while treating these factors closer to fixed effects [7]. On the other hand, when variance component are estimated and assuming an equal contribution of each locus to the variance, λ should be calculated as σ^2^_e_/(σ^2^_a_/n_snps_). In this paper, both the two options (indicated as λ_1 _and λ_2_, respectively) were tested.

For the estimation of marker effects using EBVs as dependent variable, the model for the estimation of the marker effect did not include the SEX and GEN factors.

The genomic breeding value for each of the i animals of the whole data set was then calculated as

$${\text{GEBV}}_{\text{i}}=\mu +\sum_{k=1}^{m}h{'}_{k}{\hat{b}}_{k}$$

Thus, four genomic breeding values were calculated for each animal: using phenotypes as dependent variable with variance ratio λ_1_(GEBV1) or λ_2 _(GEBV2), or using polygenic EBVs (GEBV3 and GEBV4, respectively). Accuracy of genomic selection was calculated as the correlation between GEBVs and true breeding values (TBVs).

## Results and discussion

Variance components estimated with the polygenic animal model were 1.324 and 3.142 for additive genetic (σ^2^_a_) and residual (σ^2^_e_) variance, respectively, with a resulting heritability of the trait of 0.30. Average accuracies of EBV estimation were 0.71 and 0.33 for the TRAINING and PREDICTION generations, respectively.

**Figure 1:**
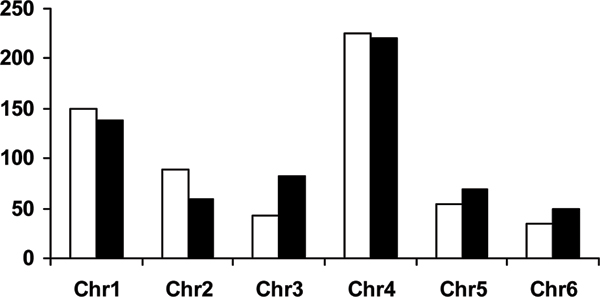
**Distribution of number of selected markers based on phenotypes (white bars) or EBVs (black bars) across the six chromosomes**.

The number of markers retained was 595 and 618 for the screening carried out on phenotypes and polygenic EBVs, respectively. In the latter case, a more stringent threshold was adopted (1.6E^-22 ^for the *P *value of the F tests) in order to retain a comparable number of markers. The largest number of significant SNPs obtained when EBVs were used could be partially explained by less noise in EBVs which already erase the fixed effects and large proportion of residuals. The number of common SNPs selected in both analyses was 411 (about 69% of the total number of retained SNPs). The average marker distance was 0.99 cM for phenotype-selected (min 0.1, max 18.6 cM) and 0.95 for EBVs-selected (min 0.1, max 19.3 cM) SNPs. The distribution of selected markers across the six chromosomes is reported in Figure 1.

Most of selected SNPs are located on chromosomes four (more than 200) and 1 (about 140). Distribution of markers selected on phenotypes (white bars) or polygenic EBVs (black bars) follow approximately the same pattern. The percentage of SNPs selected by both analyses ranged between 80% (chromosome 4) and 33% (chromosome 5). Based on variance component estimates and on the number of markers retained, variance ratios used for estimation of marker genotype effects were 2.4 (λ_1_) for GEBV1 and GEBV3, 1,412 (λ_2_) for GEBV2 and 1,466 (λ_2_) for GEBV4.

Correlations between GEBVs and TBV in the TRAINING generations are around 0.87 and 0.72 when phenotypes or EBVs are used as dependent variable (Table 1). These values are rather high, as expected, being GEBVs calculated on the animals whose phenotypes were used for estimating marker effects. In any case, the use of different variance ratios resulted in a relevant increase in the accuracy of GEBV estimation based on phenotypes. Correlations between different GEBVs and polygenic EBVs are related to the different dependent variable used. Moreover, the correlation between TBVs and polygenic EBVs corresponds to the accuracy calculated in the animal model estimation.

**Table 1:** Correlations between true breeding values (TBV), polygenic EBVs, genomic breeding values estimated using markers selected on phenotype (GEBV1 and GEBV2) and on polygenic EBVs (GEBV3 and GEBV4) for the animals of the TRAINING generations (0–3)

	TBV	EBV	GEBV1	GEBV2	GEBV3	GEBV4
TBV	*	0.70	0.78	0.87	0.74	0.72
EBV		*	0.73	0.77	0.89	0.83
GEBV1			*	0.88	0.76	0.67
GEBV2				*	0.83	0.83
GEBV3					*	0.93
GEBV4						*

Accuracies of GEBV estimation for the PREDICTION generations (Table 2) are lower (around 0.55) except for GEBV2 that shows a value still higher than 0.75, usually considered as the average GEBV accuracy in genome-wide selection schemes [3]. This result is comparable with those reported by de Roos et al. [8], in a work on actual data using polygenic EBVs as true breeding values, and of the same order of correlations reported for simulated data with similar marker density [1,9,10]. The high accuracy of GEBV2 is clearly related to the use of lower variance associated to each locus that probably prevents an over estimation of marker effects. On the other hand, the adoption of a smaller variance did not affect estimates based on EBVs. The low correlation between EBVs and TBV could be a possible explanation for these results. However, also the accuracy of about 0.55 obtained with the GEBV1, GEBV2 and GEBV4 should not be neglected, being considerably higher than that obtained with polygenic EBVs (0.33).

**Table 2:** Correlations among true breeding values (TBV), polygenic EBVs, genomic breeding values estimated using markers selected on phenotype (GEBV1 and GEBV2) and on polygenic EBVs (GEBV3 and GEBV4) for the animals of the PREDICTION generations (4–6)

	TBV	EBV	GEBV1	GEBV2	GEBV3	GEBV4
TBV	*	0.12	0.55	0.77	0.55	0.53
EBV		*	0.17	0.27	0.44	0.51
GEBV1			*	0.71	0.68	0.36
GEBV2				*	0.71	0.82
GEBV3					*	0.76
GEBV4						*

**Figure 2:**
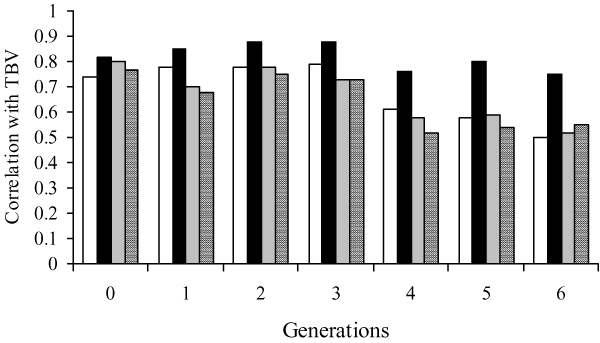
**Pattern of correlations between true breeding values and GEBV1 (white bars), GEBV2 (black bars), GEBV3 (grey bars), GEBV4 (segmented bars) across the seven generations**.

The pattern of correlations between TBVs and the different GEBVs across the seven generations considered (Figure 2) confirms the constant higher accuracy of GEBV2 and the decrease in accuracy passing from TRAINING to PREDICTION generations.

The strategy here presented for genomic breeding value estimation, that combines a pre-selection of SNPs based on least squares regression and a BLUP estimation of marker effects, gave in general poorer results in terms of accuracy of GEBV in comparison with those reported in the literature for more sophisticated methods based on Bayesian inference and on the estimation of IBD matrix. However, the GEBVs calculated using phenotypes as dependent variable and assuming an equal contribution of each marker locus to the variance of the trait showed an accuracy that is closer to the one of the best methods.

## Competing interests

The authors declare that they have no competing interests.
